# Diabetes mellitus induces a novel inflammatory network involving cancer progression: Insights from bioinformatic analysis and *in vitro* validation

**DOI:** 10.3389/fimmu.2023.1149810

**Published:** 2023-03-23

**Authors:** Yejun Tan, Jin Kang, Hongli Li, Aifang Zhong, Yaqiong Liu, Zheyu Zhang, Roujie Huang, Xin Cheng, Weijun Peng

**Affiliations:** ^1^ Department of Integrated Traditional Chinese & Western Medicine, The Second Xiangya Hospital, Central South University, Changsha, China; ^2^ School of Mathematics, University of Minnesota Twin Cities, Minneapolis, MN, United States; ^3^ Department of Experimental and Clinical Pharmacology, University of Minnesota, Minneapolis, MN, United States; ^4^ Department of Rheumatology and Immunology, the Second Xiangya Hospital of Central South University, Changsha, China; ^5^ National Clinical Research Center for Metabolic Diseases, The Second Xiangya Hospital, Central South University, Changsha, China; ^6^ Department of Emergency Medicine, The Second Xiangya Hospital, Central South University, Changsha, Hunan, China; ^7^ Centre for Research in Medical Devices, National University of Ireland Galway, Galway, Ireland; ^8^ Department of Obstetrics and Gynecology, Peking Union Medical College Hospital, Peking Union Medical College & Chinese Academy of Medical Science, Beijing, China

**Keywords:** diabetes and cancer-related genes (DCRG), diabetes-based cancer-associated inflammation network (DCIN), diabetes mellitus, inflammation, cancer

## Abstract

**Background:**

Patients with diabetes mellitus (DM) have a higher incidence of malignant tumors than people without diabetes, but the underlying molecular mechanisms are still unclear.

**Methods:**

To investigate the link between DM and cancer, we screened publicly available databases for diabetes and cancer-related genes (DCRGs) and constructed a diabetes-based cancer-associated inflammation network (DCIN). We integrated seven DCRGs into the DCIN and analyzed their role in different tumors from various perspectives. We also investigated drug sensitivity and single-cell sequencing data in colon adenocarcinoma as an example. In addition, we performed *in vitro* experiments to verify the expression of DCRGs and the arachidonic acid metabolic pathway.

**Results:**

Seven identified DCRGs, including *PPARG*, *MMP9*, *CTNNB1*, *TNF*, *TGFB1*, *PTGS2*, and *HIF1A*, were integrated to construct a DCIN. The bioinformatics analysis showed that the expression of the seven DCRGs in different tumors was significantly different, which had varied effects on diverse perspectives. Single-cell sequencing analyzed in colon cancer showed that the activity of the DCRGs was highest in Macrophage and the lowest in B cells among all cell types in adenoma and carcinoma tissue. *In vitro* experiments showed that the DCRGs verified by western bolt and *PEG2* verified by ELISA were all highly expressed in COAD epithelial cells stimulated by high glucose.

**Conclusion:**

This study, for the first time, constructed a DCIN, which provides novel insights into the underlying mechanism of how DM increases tumor occurrence and development. Although further research is required, our results offer clues for new potential therapeutic strategies to prevent and treat malignant tumors.

## Introduction

Cancer is one of the major diseases threatening people’s health worldwide (1), and 8-18% of patients with cancer also have diabetes as a coexisting illness (2). Diabetes Mellitus (DM) is associated with higher cancer risk, peaking approximately eight years after diagnosis (3). Epidemiological findings have shown up to a two-fold increase in the risks of colorectum, liver, and pancreas cancers among patients with DM (4). In addition, the incidence of oral cancer in patients with DM is also significantly increased (5, 6). Between 1988-1994 and 2010-2015, cancer mortality rates dropped in the United States but remained about 30% higher in adults with DM compared to those without DM (7), suggesting that effective control of DM could decrease the healthcare burden and improve the quality of life (8). Additionally, it has been reported that managing hyperglycemia and insulin resistance in patients with DM who are also suffering from cancer may improve their overall quality of life. Given the substantial medical burden of these diseases worldwide, understanding the association between cancer and DM might be essential to public health (9, 10).

Preliminary investigations to understand the mechanisms underlying the relationship between DM and cancer demonstrated the involvement of apoptotic and proliferation pathways (10, 11). They showed the involvement of factors such as hyperglycemia, hyperinsulinemia, insulin-like growth factor 1 (IGF-1), oxidative stress, and sex hormones. The association between DM and cancer may be causal (resulting from hyperinsulinemia or hyperglycemia) or confounding (resulting from common risk factors such as obesity) (12). Despite the prevalence of cancer and DM, the extent to which DM affects cancer remains unclear. Investigating the correlation between DM and cancer will contribute to a better understanding of cancer’s pathogenesis, as well as provide a better reference for individualized treatment of diabetic tumors.

Emerging evidence suggests that chronic inflammation is a crucial link between DM and cancer (13). Diabetes mellitus is associated with inflammation-related insulin secretory defects (14). Chronic inflammation, characterized by high levels of oxidative stress and reactive oxygen species (ROS), activation of pro-inflammatory pathways, and abnormal adipokine production, may develop a microenvironment that promotes tumor cell growth, facilitates metastasis, increases angiogenesis, and impairs natural killer cells and macrophages (15). Cross-talk between cancer and DM is also influenced by oxidative stress. Hyperglycemia could increase the production of superoxides (16). Additionally, insulin could stimulate the production of ROS (17). Strong evidence suggests that oxidative stress influences the expression of several genes and signal transduction pathways that play an essential role in tumorigenesis (18). As a result of cytokine-dependent activation of nuclear factor (NF)-kB pathways, ROS interferes with cell proliferation and apoptosis (19).

Studies show that NF-kB is hyperactivated in colorectal cancer, breast cancer, blood neoplasms, and pancreatic cancer cell lines. Inflammation facilitates tumor development, progression, and treatment resistance. In contrast, acute inflammatory responses often stimulate dendritic cells (DCs) to mature and present antigens, resulting in anti-tumor immune responses (20) (21).

To explore the interaction between DM and cancer, we screened seven diabetes and cancer-related genes (DCRGs) using public databases and constructed a diabetes-based cancer-associated inflammation network (DCIN). We analyzed the role of the seven DCRGs in cancer from different perspectives. Drug sensitivity and single-cell sequencing data were analyzed using colon adenocarcinoma (COAD). The expression of DCRGs and the arachidonic acid metabolic pathway was further verified *in vitro*. Therefore, for the first time, our study constructed a diabetes-based cancer-associated inflammation network, offering novel insights into the underlying mechanism of DM increasing tumor occurrence and development. Although further research is still required, our results hint toward potential therapeutic strategies in preventing and treating malignant tumors.

## Materials and methods

### Screening for genes associated with DM and various cancers

Epidemiological findings have shown a two-fold increase in the risks of colorectum, liver, and pancreas cancers among patients with DM (4). Therefore, DisGeNET (https://www.disgenet.org/) was used to explore genes related to DM, as well as pancreatic, liver, and colon cancers (22). These genes were filtered using a correlation score greater than 0.1 (*r* > 0.1). The intersection of genes related to these four diseases was taken from DisGeNET. Similarly, GeneCards (https://www.genecards.org/) was also used to search for genes associated with DM, pancreatic cancer, liver cancer, and colon cancer. These genes were filtered using a correlation score greater than 1 (*r* > 1). The intersection of genes related to these four diseases was taken from GeneCards. The “ggplot2” software package was used to visualize the intersection in Venn diagrams.

### Data downloading and preprocessing

Gene expression profiles, phenotypic information, and survival data of 33 TCGA tumor samples and adjacent tissues (11057 samples in total) were downloaded from the UCSC Xena database (http://xena.ucsc.edu/). The gene expression profiles were set in Fragments Per Kilobase Per Million (FPKM) and HTseq-counts format. Demographic, tumor information, and follow-up data were also extracted from the database for all patients. Subsequently, the expression profiles of *PPARG*, *MMP9*, *CTNNB1*, *TNF*, *TGFB1*, *PTGS2*, and *HIF1A* were extracted from 33 types of TCGA tumor and adjacent tissue samples for further analysis.

### Differential and co-expression analysis

The expression of the seven DCRGs between tumor and normal tissues was assessed using the Wilcox test and visualized using “ggplot2” for all the TCGA tumor types analyzed. The differential expression of the seven DCRGs between tumor and normal tissues across cancers was presented as a log2 fold change in the heatmap. Co-expression between these seven DGRGs at the transcriptional level was analyzed by the “corrplot” software package. In addition, the STRING database (https://string-db.org/) was used to construct a protein-protein interaction (PPI) network among these genes. The interactions between DCRGs were obtained from the string database, and a medium confidence level (0.40) was chosen to construct the protein-protein interaction network.

### Clinical correlation analysis

Kaplan-Meier plots of DCRGs were generated using the R package to analyze the differences in overall survival outcomes between patients with high and low expression of the seven DCRGs. The phenotype and survival data of 33 TCGA cancer types obtained from the UCSC Xena database were analyzed. These DCRGs were divided into high and low-expression groups according to the median expression level for survival analysis. The software packages “Survival” and “SurvMiner” were used to plot survival curves. The “ggplot2” software package created a heatmap to represent clinical correlations; the heatmap of clinical correlations (overall survival) for DCRGs displayed only the statistically significant tumor types (∗∗ *P*<0.001; ∗∗ *P*<0.01; ∗ *P*<0.05). Red squares indicate high gene expression associated with poor prognosis, while blue squares indicate low gene expression associated with poor prognosis. In addition, the hazard ratios of DCRGs in each TCGA tumor type were obtained using the Cox proportional hazards regression. The Cox proportional hazards model was constructed using “survival” and “Forestplot” software packages to determine the pan-cancer relationship of the seven DCRGs with overall survival. Furthermore, the expression of these seven DCRGs was evaluated in patients with COAD with different stages. *P* < 0.05 were considered significant.

### Gene set variation analysis of DCRGs

In order to investigate the potential pathways of these DCRGs, we performed GSVA analysis on the pathways of the seven DCRGs across cancers. Gene sets for GSVA were obtained from the MSigDB database (c2. Cp. Kegg. V7.1. Symbols. gmt). Firstly, the “GSVA” software package was used to generate GSVA scores for pan-cancer expression profiles of all seven genes. Then, the “limma” software package was used to analyze the differences between pan-cancer tumors and paracancerous tissues. The pathways with | t-value of GSVA score | > 2 were considered significant. Finally, the “ggplot2” software package was used to visualize the differences, and R software was used to count the significant pathways across various types of cancers. 

### Relationship between DCRGs expression and tumor immune cell infiltration

To better understand the relationship between DCRGs and immune cells, the association between the gene expression levels of the seven DCRGs and the infiltration levels of 22 immune-related cells was estimated. CIBERSORT (https://cibersort.stanford.edu/) was used to estimate the extent of immune cell infiltration across cancer samples. Finally, “ggplot2”, “ggpubr”, and “ggExtra” software packages were used to assess the correlation between the levels of the seven DCRGs and each immune cell infiltration in cancer (*P* < 0.05 was considered significant).

### Immune subtype analysis

Tumor immune microenvironment (TIME) has therapeutic and prognostic significance in anti-tumor therapy. Studies have identified six immune subtypes of tumor types based on five representative immune signatures, which include wound healing (C1), IFN-γ dominant (C2), inflammatory (C3), lymphocyte depleted (C4), immunologically quiet (C5), and TGF-β dominant (C6) (22). Differential expression analysis was performed using the Kruskal test to understand the mRNA expression levels of DCRGs in the six different immune subtypes of tumor types. Furthermore, the mRNA expression levels of the seven DCRGs were analyzed in COAD.

### Stemness indices and TIME across cancers

We assessed Stemness indices and ESTIMATE scores (Estimation of STormal and Immune cells in MAlignant Tumor tissues using Expression data) in pan-cancer (23). The ESTIMATE score is calculated based on gene expression characteristics and can reflect tumor purity with good prediction accuracy. Therefore, Spearman correlation analysis was performed between the expression levels of the seven DCRGs genes and matrix scores by “Estimate” and “limma” packages.

In order to further analyze the association between DCRGs and pan-cancer stemness features, the stemness index of tumor samples was calculated using a one-class logistic regression (OCLR) algorithm. Subsequently, the Spearman correlation analysis was performed based on gene expression and stemness score (24). Here, two types of dryness indices were obtained: DNA methylation-based stemness score (DNAss) and mRNA expression-based stemness score (RNAss).

### Mutations in the seven DCRGs across cancers

The samples for pan-cancer analysis of whole genomes (ICGC/TCGA, Nature 2020) were obtained from cBioPortal (http://www.cbioportal.org/). Based on previous studies, a CNV value of 2 was considered as amplification and a value of -2 was considered as deep deletion. The CNV ratios of DCRGs for each tumor type were calculated for each tumor type (25). In addition, the overall cellular landscape of changes in the OncoPrint plot was generated using the R software package “ComplexHeatmap”.

### Correlation of the expression levels of the seven DCRGs with TMB and MSI

Tumor mutation burden (TMB) is a quantifiable biomarker of immune response that reflects the number of mutations in tumor cells (26). Microsatellite instability (MSI) is caused by major molecular response (MMR) deficiency and is associated with the prognosis of patients (27). TMB and MSI are intrinsically related to immune checkpoint inhibitor sensitivity. The correlation between the expression levels of the seven DCRGs with TMB and MSI was investigated. A Perl script was used to calculate the TMB score, corrected by the total length of exons. MSI scores were determined for all samples based on somatic mutation data downloaded from TCGA. Spearman correlation coefficients were used to analyze the relationship of DCRGs expression with TMB and MSI. The result was displayed as a heatmap generated by the “ggplot2” software package.

### Correlation analysis between the seven DCRGs and immune checkpoint-related genes in COAD

Due to the high frequency of mutations of the seven genes in COAD, it was selected for further analysis. R software was used to analyze the correlation between the seven DCRGs and 46 immune checkpoint-related genes in COAD. The result was also visualized as a heatmap by the “ggplot2” software package.

### Pan-cancer drug sensitivity analysis

CellMiner is a web-based tool containing genomic and pharmacological information for investigators to utilize transcript and drug response data from the NCI-60 cell line set compiled by the National Cancer Institute. RNA-seq spectrum data of the seven DCRGs and their pharmacological activity were collected from the CellMiner database (https://discover.nci.nih.gov/cellminer/). The “Impute” software package was used to preprocess the raw data. The correlation between the transcriptional expression of the seven DCRGs and compound sensitivity was investigated using Pearson correlation analysis. When the *P* was less than 0.05 and the correlation coefficient was greater than 0.3, the relevant DCRGs were considered sensitive to the corresponding chemotherapeutic drugs.

### Validation of DCRGs in single-cell transcriptome sequencing data

To validate the function of the seven DCRGs in colon cancer, single-cell sequencing data of colon cancer were analyzed. The GSE161277 dataset was downloaded from the Gene Expression Omnibus (GEO) database, and the raw data for each sample was processed using the “Seurat” software package in R (28). Genes expressed by fewer than three cells in the sample, genes with fewer than 200 expressed genes, and mitochondrial gene content that exceeded 25% of the total unique molecular identifier (UMI) count were excluded. The data were standardized using the default method, and the most variable genes were identified and selected using the FindVariableFeatures method. These genes were then centered and scaled before performing a principal component analysis to identify clusters of cells. Stromal and immune cells were annotated based on specific markers from previous studies.

The activity of DCRGs was calculated in the single-cell dataset using the AUCell software package, and the scores were visualized using ggplot2 to create UMAP plots and boxplots. Additionally, Kruskal-Wallis tests were conducted between the adenoma, blood, carcinoma, normal, and para-cancer groups using the ggpur software package. The AUCell scores were considered to be different between groups when the *P*-value was less than 0.05. “Gene set variation analysis was performed using the GSVA software” package on the cell type with the highest activity of DCRGs to investigate differentially activated metabolic pathways. The subpopulation of cells with the highest AUC values for all DCRGs was also analyzed using a pseudo-time sequence, and cells were sorted based on subpopulation markers obtained from the previous analysis. The relationship between overall cellular expression changes was evaluated, and a single-cell pseudo-time trajectory was generated using the monocle software package in R. Finally, the mean expression of the seven DCRGs was calculated, and the expression values of the DCRGs for each developmental stage were obtained.

### Cell culture

The SW480 cells were grown in Dulbecco’s modified Eagle’s medium (DMEM, GENVIEW, GD3103) supplemented with 10% fetal bovine serum (FBS) and 1% penicillin/streptomycin (PS). The NCM460 cells were grown in 1640 medium (GENVIEW, GR3101) with 10% FBS and 1% PS (NCM Biotech, C100C5). Cells were grown at 37 °C in an atmosphere of 5% CO2 and 95% relative humidity, and the medium was changed every 2 days. The NCM460 cells were grown in 1640 medium (GENVIEW, GR3101) with 10% FBS and 1% PS (NCM Biotech, C100C5). Cells were grown at 37 °C in an atmosphere of 5% CO2 and 95% relative humidity, and the medium was changed every 2 days. When cells reached approximately 90% confluency, they were detached using 0.1% trypsin-ethylenediaminetetraacetic acid (NCM Biotech, C100C1) and seeded in a 6-well plate. Subsequently, the cells were cultured with high glucose (HG, 50 mmol/l) or normal glucose (NG, 5.5 mmol/l) for 2 days at 37°C.

### Western blot analysis

The total proteins of SW480 and NCM460 cells were prepared using RIPA buffer containing protease and phosphatase inhibitors. A BCA protein assay kit was used to measure protein concentrations. 20 µg proteins were loaded per lane, separated by electrophoresis, and then transferred to polyvinylidene fluoride (PVDF) membranes (C3117, Millipore). The membrane was blocked and then incubated for 1 h with β-actin (1:100000; ABclonal, AC026), PPAR gamma (1:5000; Proteintech, 16643-1-AP), MMP9 (1:1000; Proteintech, 10375-2-AP), TNF alpha (1:3000; Proteintech, 60291-1-IG), TGF beta1 (1:2000; Proteintech, 21898-1-AP), COX2 (1: 500; Proteintech, 27308-1-AP), HIF1A (1:2500; Proteintech, 20960-1-AP) and Beta-catenin (1:10000; Proteintech, 51067-2-AP). Immunoblot analysis was performed with horseradish peroxidase (HRP)-conjugated anti-mouse antibodies or anti-rabbit antibodies (1:5000; ZSGB-BIO, ZB-5301, and ZB-5305) and developed with the ECL kit (Beyotime Biotechnology, P0018FM). The level of β-actin was used as a loading control, and the ratios of the gray value of the target protein bands to the gray value of the corresponding internal control bands were defined as the expression level of the target protein.

### Enzyme-linked immunosorbent assay

After treatment in accordance with the conditions described above, the concentration of Leukotriene B4 (LT-B4), Leukotriene C4 (LT-C4), and Prostaglandin E2 (PG-E2) was tested using an enzyme-linked immunosorbent assay (ELISA) kit. These ELISA kits included Human Leukotriene B4, LT-B4 ELISA Kit (CSB-E08033h), Human Prostaglandin E2, PG-E2 ELISA Kit (CSB-E07965h), and Leukotriene C4 Assay Kit (H556-1). In brief, cells were seeded into 6-well plates, followed by incubation for 2 days at 37°C. Then, the cultivating supernatant was collected, and the presence of these factors was determined by ELISA following the manufacturer’s protocols.

## Results

### Differential expression analysis and co-expression analysis of DCRGs

**Figure 1:**
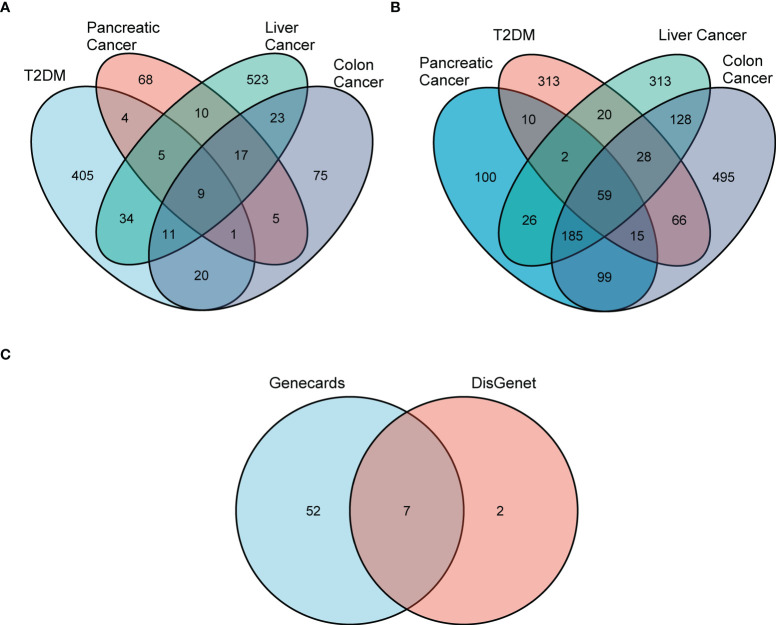
Screening for differential DCRGs. **(A)** Venn diagram showing nine intersection genes of five diseases, T2DM, pancreatic cancer, liver cancer, and colon cancer, based on DisGeNET (r > 0.1). **(B)** The 59 intersection genes of the five diseases based on GeneCards (r > 0.1). **(C)** The seven DCRGs obtained by the intersection of DisGeNET and GeneCards.

**Figure 2:**
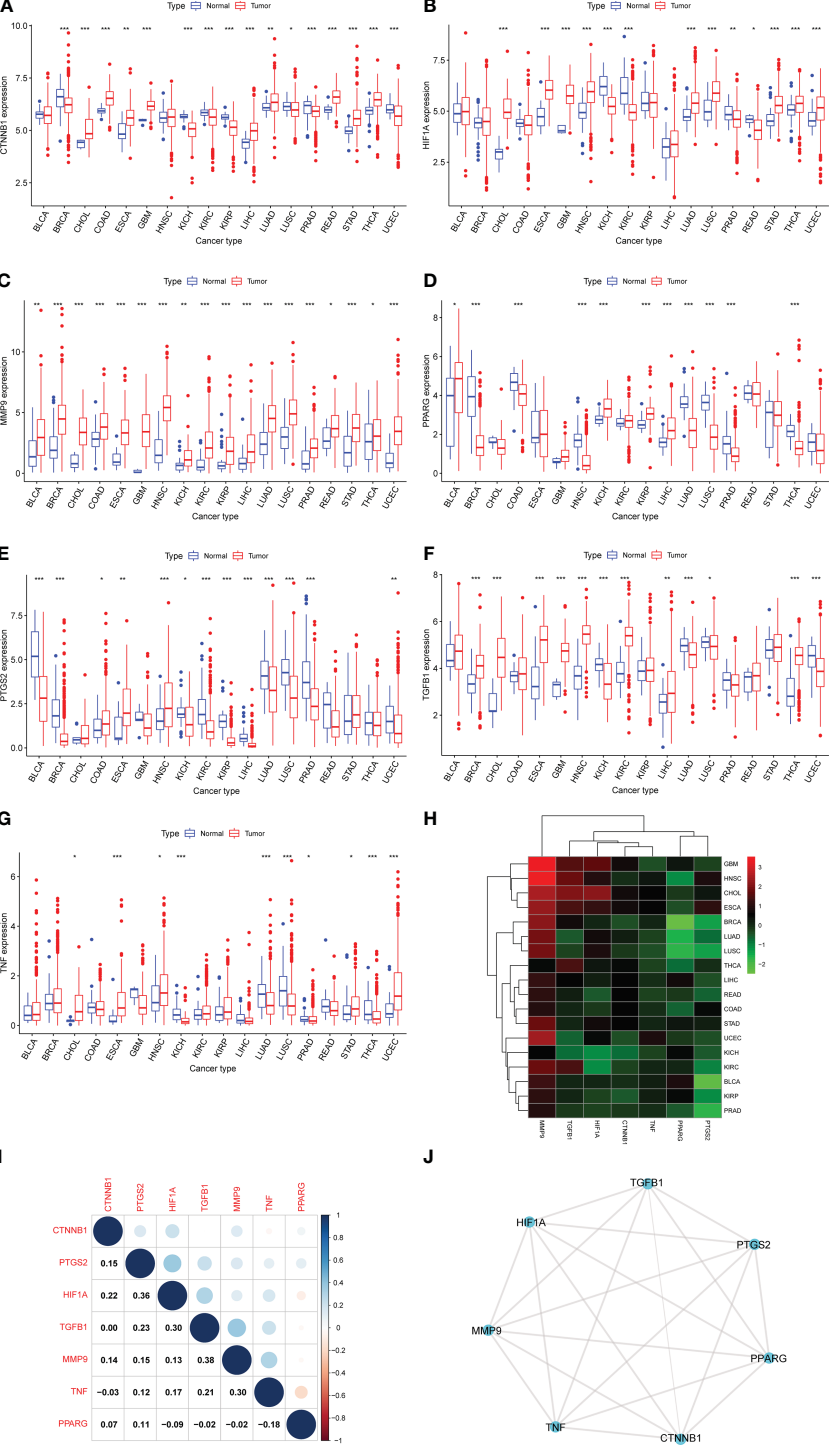
Differential expression analysis. **(A–G)** The box plots showing differential expression of the seven DCRGs in normal and tumor tissues (∗∗∗ *P* < 0:001; ∗∗ *P* < 0.01; ∗ *P* < 0.05). **(H)** The heatmap showing the transcriptional level of the seven DCRGs between normal and tumor tissues in various cancers. The gradient colors represent the log Fold Change (logFC) value. **(I)** The heatmap shows the pairwise correlation of the seven DCRGs. **(J)** The protein-protein interaction network of the seven DCRGs.

First, we used DisGeNET to identify gene sets related to DM, pancreatic cancer, liver cancer, and colon cancer (*r* > 0.1) and obtained nine intersection genes. Then we used GeneCards to search for DM, pancreatic cancer, liver cancer, and colon cancer-related genes (*r* > 1) and obtained 59 intersection genes. Finally, by taking the intersection of the nine genes from DisGeNET and 59 genes from GeneCards, we identified seven DCRGs: *PPARG*, *MMP9*, *CTNNB1*, *TNF*, *TGFB1*, *PTGS2*, *HIF1A* (**Figures 1A–C**). The expression of DCRGs varied among different tumors. *MMP9* showed high expression in all tumors except THYM, *TNF* displayed high expression in most tumors, and *PTGS2* exhibited low expression in most tumors. Meanwhile, the DCRGs exhibited no statistical differences in MESO and UVEM (**Figures 2A–H**, **Supplementary Figure S1**). Interestingly, the correlation analysis revealed a correlation between *HIF1A* and *PTGS2* (*r*=0.36), *TGFB1* and *HIF1A* (*r*=0.3), *MMP9* and *TGFB1* (*r*=0.38), *TNF* and *MMP9* (*r*=0.3, **Figure 2I**). Meanwhile, the seven DCRGs were used to constitute a protein-protein interaction network (**Figure 2J**). As each DCRG was associated with inflammation, we defined the network as DCIN.

### Clinical correlation analysis

**Figure 3:**
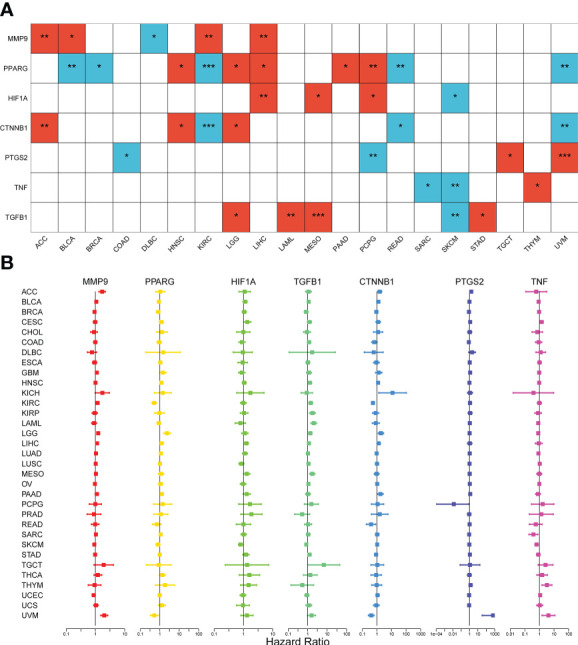
Survival analysis of the seven DCRGs across cancers. **(A)** The heatmap of clinical correlation analysis (overall survival) of DCRGs (∗∗∗ *P* < 0:001; ∗∗ *P* < 0.01; ∗ *P* < 0.05). Only tumor types with statistical significance are shown. The red square indicates high gene expression is associated with a worse prognosis; the blue square indicates low gene expression is associated with a worse prognosis. **(B)** Cox proportional hazard analyses illustrate the hazard ratios (HRs) of the seven DCRGs in 33 TCGA tumors. DCRGs with HR > 1 were regarded as risk factors, while DCRGs with HR < 1 were regarded as protective factors.

To investigate the association between expression levels and the prognosis of the seven DCRGs, we performed an overall survival analysis of 33 cancers. Patients were divided into high-expressing and low-expressing groups according to the median gene expression levels. High and low expressions of the seven DCRGs were associated with overall survival in multiple cancers (**Supplementary Figure S2**, **Figure 3**). The Kaplan-Meier survival analysis revealed that patients with KIRC, READ, and UVM who had high *CTNNB1* levels had better overall survival (OS) rates (*P*<0.001, *P*=0.024, and *P*=0.002, respectively). However, patients with ACC, HNSC, and LGG who had high *CTNNB1* were associated with poor OS (*P*=0.004, *P*=0.046, and *P*=0.0179, respectively). Similarly, patients with SKCM who had high *HIF1A* levels had better survival rates (*P*=0.026), but patients with LIHC, MESO, and PCPG who had high *HIF1A* were associated with poor OS (*P*=0.004, *P*=0.03, and *P*=0.045, respectively). Additionally, patients with DLBC who had high levels of *MMP9* had better survival rates (*P*=0.017), while high *MMP9* in patients with ACC, BLCA, KIRC, and LIHC was associated with poor OS (*P*=0.003, *p*=0.03, *P*=0.001, and *P*=0.009, respectively). Furthermore, patients with BLAC, BRCA, KIRC, READ, and UVM who had high *PPARG* levels had longer OS rates (*P*=0.001, *P*=0.04, *P*<0.001, *P*=0.003, and *P*=0.003, respectively). However, high *PPARG* in patients with HNSC, LGG, LIHC, PAAD, and PCPG were associated with poor OS (*P*=0.043, *P*=0.041, *P*=0.014, *P*=0.023, and *P*=0.004, respectively). Patients with COAD and PCPG who had high *PTGS2* expression had longer OS rates (*P*=0.028 and *P*=0.003, respectively), whereas high *PTGS2* expression in patients with TGCT and UVM was associated with poor OS (*P*=0.043 and *P*=0.004 respectively). Similarly, patients with SKCM who had high levels of *TGFB1* had longer survival rates (P=0.008), while high *TGFB1* in patients with LAML, LGG, MESO, and STAD was associated with poor OS (*P*=0.003, *P*=0.03, P< 0.001, and *P*=0.027 respectively). Lastly, patients with SKCM and SARC who had high *TNF* expression had longer OS rates (*P*=0.003 and *P*=0.013, respectively), whereas high *TNF* expression in THYM patients was associated with poor OS (*P*=0.018).

Moreover, we used Cox proportional hazard regression to examine the prognostic effects of the DCRGs across 33 TCGA tumors (**Figure 3**). The results showed that *CTNNB1* was a protective factor for KIRC (*P*<0.001), READ (*P*=0.014), and UVM (*P*<0.001) and a risk factor for ACC (*P*=0.007), and LGG (*P*=0.003). *HIF1A* was a protective factor for SKCM (*P*=0.003) and a risk factor for LIHC (*P*=0.006) and MESO (*p*=0.04). *MMP9* was a protective factor for SKCM (*P*=0.009), UCEC (*P*=0.04) and a risk factor for ACC (*P*<0.001), BICA (*P*=0.048), KIRC (*P*<0.001), LGG (*P*<0.001), UVM (*P*<0.001), GBM (*P*=0.03) and LIHC (*P*=0.01). *PPARG* was a protective factor for BLCA (*P*=0.01), KIRC (*P*<0.001), and UVM (*P*=0.005) and a risk factor for HNSC (*P*=0.004), LGG (*P*<0.001), LIHC (*P*=0.009), and PAAD (*P*=0.003). *PTGS2* was a protective factor for PCPG (*P*=0.04) and a risk factor for ACC (*P*<0.001), CESC (*P*=0.01), PAAD (*P*=0.007), and UVM (*P*<0.001). *TGFB1* was a protective factor for SKCM (*P*=0.009) and a risk factor for HNSC (*P*=0.03), KIRC (*P*=0.001), KIRP (*P*<0.001), LAML (*P*<0.001), LIHC (*P*=0.03), LGG (*P*=0.003), MESO (*P*<0.001), SKCM (*P*=0.01), TGCT (*P*=0.047), and STAD (*P*=0.03). *TNF* was a protective factor for SARC (*P*=0.007) and SKCM (*P*<0.001) and a risk factor for CESC (*P*=0.004), UVM (*P*=0.006), and THYM (*P*=0.003). Notably, the prognostic effects of DCRGs varied across different tumors, with high expression levels of the same DCRG being associated with either a protective or a risk factor for OS in different cancer types.

### Gene set variation analysis of DCRGs across cancers

To investigate the involvement of these seven DCRGs in cancer-related pathways and the pathways related to DM and carcinogenesis, we performed gene set variation analysis (GSVA) on the DCRGs across cancers. We considered pathways with | t value of GSVA score | > 2 between tumor and control samples as significant. Among them, the metabolism-related pathways, such as PPAR, mTOR, VEGF, and arachidonic acid signaling pathways, appeared multiple times in various cancers. Additionally, type I and type II DM also appeared in various cancer pathways (**Supplementary Figure S2**), indicating that these seven DCRGs are closely related to DM and metabolism.

### Immune cell infiltration analyses of DCRGs across cancers

We next examined the association between the seven DCRGs and the infiltration levels of 22 immune-related cells. Our data showed that in most cancers, the level of immune cell infiltration was significantly correlated with the expression of the DCRGs (|*r*| ≥ 0.5, *P* < 0.01). Among them, *MMP9* was associated with macrophage M0 in 12 tumor types, *PTGS2* was associated with six immune cell types among six tumor types, and *TNF* with seven immune cell types in five tumor types (**Supplementary Figure S3**).

### Immune subtype analysis

**Figure 4:**
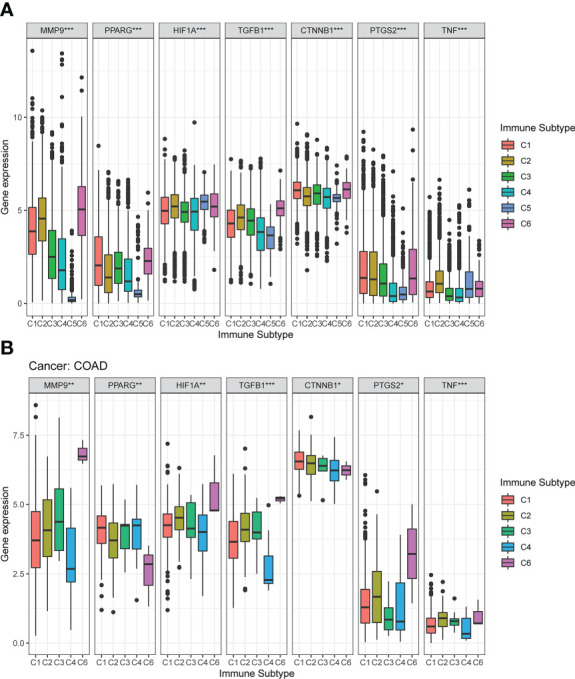
Expression pattern of the seven DCRGs in six different immune subtypes. **(A)** Transcriptional expression of the seven DCRGs in C1-C6 immune subtypes across all TCGA cancers. **(B)** Box plots showing the expression level of the DCRGs in the immune subtypes in COAD (∗∗∗ *P* < 0.001; ∗∗ *P* < 0.01; ∗ *P* < 0.05).

The mRNA expression of the seven DCRGs were analyzed using the Kruskal test in six immune subtypes across 33 TCGA tumor types. Results showed that the expression of these seven DCRGs varied significantly across C1-C6 subtypes in different cancer types (*P*<0.001). Specifically, *CTNNB1* exhibited the highest expression level in the C1-C6 immune subtypes (**Figure 4A**). Similarly, the seven DCRGs were differentially expressed in the C1-C6 immune subtypes of COAD (*P*<0.05), with *CTNNB1* exhibiting the highest expression compared to the other six DCRGs. (**Figure 4B**). Moreover, we found that C6 was the immune isoform with the highest expression level in the remaining DCRGs, except for CTNNB1.

### Stemness indices and tumor microenvironment

The matrix fraction of the TCGA cancer samples was calculated by applying the ESTIMATE algorithm. Spearman correlation analysis was used to describe the correlation between the expression levels of the DCRGs and the pan-cancer matrix score. We found that the expression of *MMP9*, *TGFB1*, and *TNF* was positively correlated with immune and stromal scores (*P* < 0.05, *r* > 0.5, **Supplementary Figures S4A, B**). To analyze the correlation between the DCRGs and pan-cancer stemness characteristics, we calculated the stemness index of the TCGA tumor samples using a Class I logistic regression (OCLR) algorithm. Subsequently, spearman correlation analysis was performed based on gene expression and stemness score. Two types of dryness indices were calculated: DNA methylation-based stemness score (DNAss) and mRNA expression-based stemness score (RNAss). The correlation between the two stemness indexes and the expression levels of the seven DCRGs varied in the TCGA tumors. DNAss exhibited a strong correlation between OV and *TGFB1*, THYM and *PTGS2*, and TCGT and *CTNNB1*. RNAss demonstrated that *TGFB1* was negatively correlated with many cancer types, while *CTNNB1* was negatively correlated with THYM (**Supplementary Figures S4C, D**).

### Mutations in the seven DCRGs across cancers

**Figure 5:**
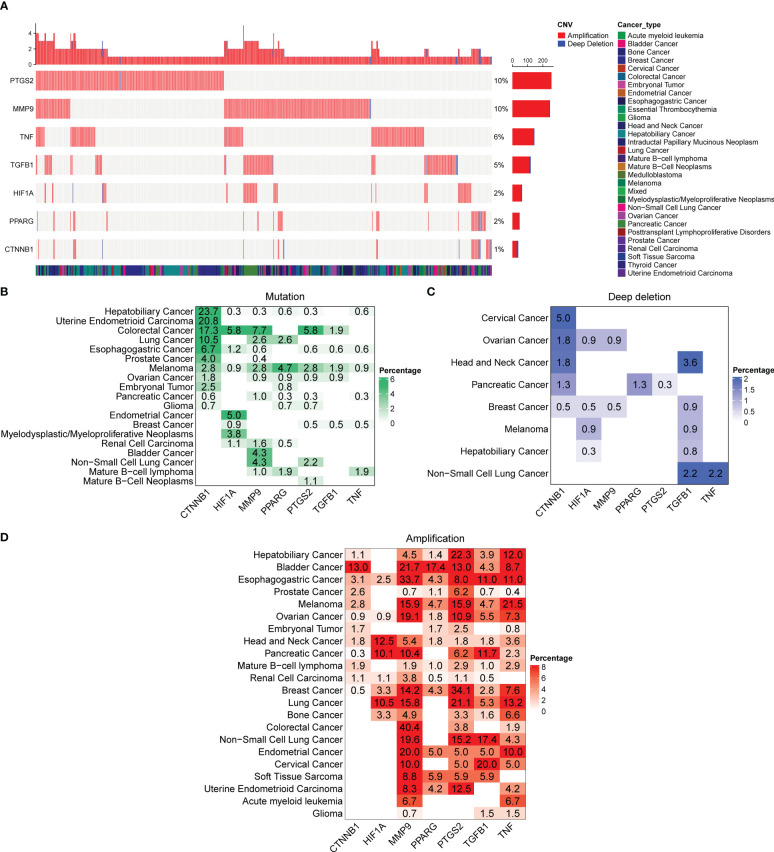
Alterations in the DCRGs in cancer based on pan-cancer analysis of whole genomes. **(A)** Copy number variation (CNV) landscape of DCRGs in various tumors. Each row represents a gene, and each column represents a patient. The CNV frequency of DCRGs is shown. The figure only represents patients with DCRG alterations. The mutation rate of each gene is shown in the labels on the right. **(B–D)** Frequency distribution of mutation **(B)**, deep deletion **(C)**, and amplification **(D)** in different cancer types. The numbers in the figure represent the specific mutation rate, and the color intensity is proportional to the frequency.

To better understand the genomic alterations of DCRGs in tumors, we analyzed copy number variation (CNV) data of 2,922 samples from the Pan-cancer analysis of whole genomes (ICGC/TCGA, Nature 2020) and depicted the resulting CNV landscape. Our findings indicated that *PTGS2* and *MMP9* had a higher CNV frequency at 10%, followed by *TNF* and *TGFB1* at 6% and 5%, respectively (**Figure 5A**). Amplifications were the most common CNV types observed for DCRGs. We then described the somatic changes in each DCRG for each tumor to determine the situation across different tumor types with varying mutation patterns. Notably, DCRGs showed a high mutation frequency in colorectal cancer and melanoma, with *CTNNB1* having the highest mutation frequency in hepatobiliary cancer (23.7%) and uterine endometrioid carcinoma (20.8%) (**Figure 5B**). In colorectal cancer, *HIF1A*, *MMP9*, and *PTGS2* had the highest mutation frequency at 5.8%, 7.7%, and 5.8%, respectively, while in melanoma, *PPARG* and *TGFB1* had the highest mutation frequency at 4.7% and 1.9%, respectively. Additionally, *CTNNB1* had the highest deep deletion rate across pan-cancer at 5%, followed by *TGFB1* at 3.6% in head and neck cancer and 2.2% in non-small cell lung cancer, and *TNF* and *PPARG* in pancreatic cancer with rates of 3.6% and 1.3%, respectively, (**Figure 5C**). The deep deletion rate of other DCRGs in cancers was less than 1%. *PTGS2* and *MMP9* had the highest amplification rates, with *DCRGs* amplified in bladder cancer, esophagogastric cancer, ovarian cancer, head and neck cancer, and breast cancer (**Figure 5D**). Notably, *MMP9* had a staggering amplification frequency of 40.4% in colorectal cancer and 33.7% in esophagogastric cancer, while *PTGS2* had an amplification rate of 34.1% in breast cancer.

### Association of the DRCGs with the immunomodulators, TMB and MSI

We then investigated whether the expression levels of the seven DCRGs correlated with TMB and MSI, both of which are intrinsically related to immune checkpoint inhibitor sensitivity. The results showed that, except for KICH, READ, CHOL, TGCT, and LAML, the TMB of the remaining 28 tumors was correlated with the expression of at least one of the seven DCRGs (**Supplementary Figure S5A**). Meanwhile, except for CHOL, OV, UCS, and LAML, the MSI of the other 29 cancers was correlated with the expression of at least one of the seven DCRGs (**Supplementary Figure S5B**).

### Seven DCRGs predict the response to cancer immunotherapy

Due to the high frequency of mutations of the seven DCRGs in COAD, they were selected for further analysis. We analyzed the association of the seven DCRGs with multiple cancers and 46 immune checkpoint-related genes in COAD. All 46 immune checkpoint-related genes were associated with at least one of the seven DCRGs. Among them, *CD274* (PD-L1) was correlated with six genes except for *MMP9* (**Supplementary Figure S5C**).

### Drug sensitivity analysis across cancers

To analyze the potential effect of the DCRGs on drug response, we performed a Pearson correlation analysis between the transcriptional expression of the seven DCRGs in the NCI-60 cell line and the drug activity of 263 anti-tumor drugs retrieved from the CellMiner database. Scatterplots (**Supplementary Figure S6**) sorted by value exhibited significant correlations between drug sensitivity and gene expression. Notably, Nelarabine and *TNF* showed a high correlation (|*r*|=0.747). Together, our results showed that multiple drugs were sensitive to the seven DCRGs (*p*<0.001, |*r*|>0.4).

### Validation of DCRGs in single-cell data set of colon cancer

**Figure 6:**
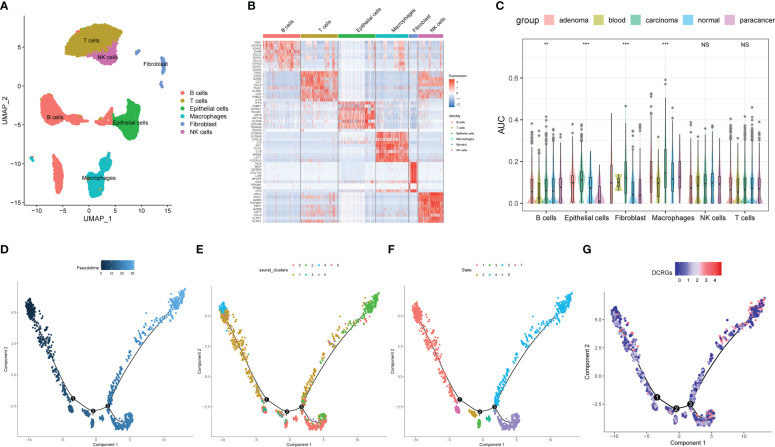
Analysis of single-cell RNA sequencing of COAD. **(A)** UMAP plot of the cell clusters of GSE161277. **(B)** Top 10 marker genes among the eight cell clusters. **(C)** AUC scores of the DCRGs among each cell type. **(D)** The cell differentiation timeline, where deeper colors represent earlier developmental stages. **(E)** The distribution of different clusters of macrophages in the cell trajectory curve. **(F)** The distribution of different states of macrophages on the cell trajectory curve. **(G)** Distribution of the average expression of DCRGs on the cell trajectory curve. ** P < 0.01,*** P < 0.001.

For the evaluation of DCRG activity in single cells of colon cancer, 12 single-cell samples were included, consisting of four adenoma, one blood, four carcinoma, one para-cancer, and three normal tissues (28). Six cell types were identified according to the previous studies (**Figure 6A**). **Figure 6B** shows the top 10 marker genes for each cell type (**Figure 6B**). The AUCell analysis showed that macrophages had the highest AUC values for the DCRGs in carcinoma and adenoma, while the lowest values were observed in epithelial cells in para-cancer tissues (**Figure 6C**, **Supplementary Figure S7C**). In fact, the AUC values of DCRGs differed between adenoma, blood, carcinoma, normal, and para-cancer groups in B cells, epithelial cells, fibroblasts, and macrophages (*P*<0.05). Moreover, GSVA results indicated that the arachidonic acid metabolism pathway was upregulated (t > 2) in the adenoma and carcinoma tissues compared to the normal tissue. (**Supplementary Figures S7A**, **6B**). Furthermore, pseudo-time sequence analysis was performed to confirm the developmental stages of the macrophage subpopulations. **Figure 6D** illustrates the cell differentiation timeline, where deeper colors represent earlier developmental stages. The results show that the seven clusters can be roughly divided into seven differentiation states (**Figures 6E–G**). Among these, clusters 4, 1, and 6 were in the early developmental stage, while cluster 2 was in the late developmental stage. The mean expression of DCRGs was higher in cluster 1 and highest in cluster 2, indicating that DCRGs have higher average expression values in the early and late stages of macrophage development, potentially linked to macrophage differentiation.

### Verification of the seven DCRGs and arachidonic acid pathway *in vitro*

**Figure 7:**
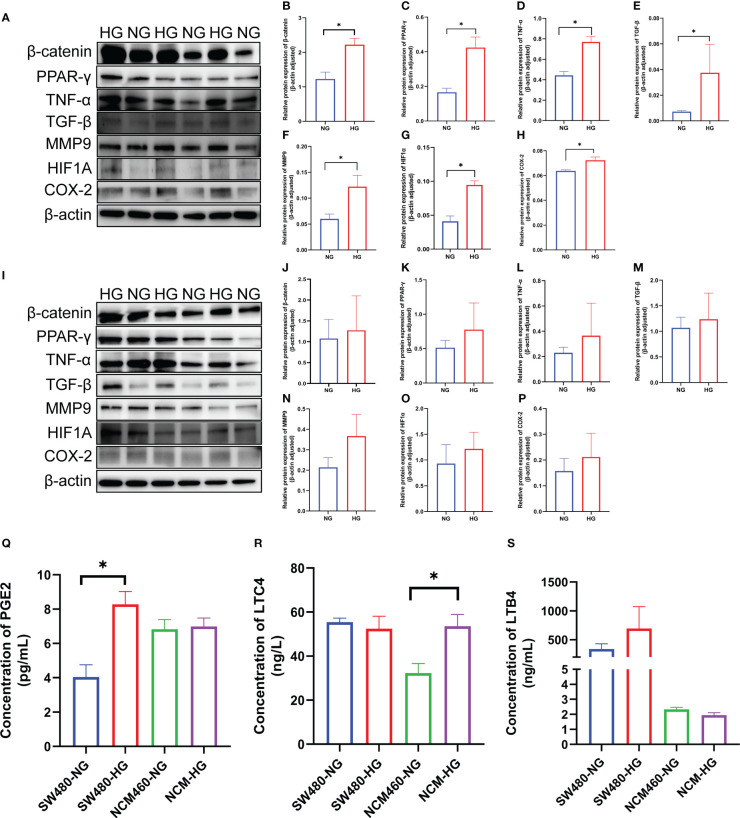
Verification of the seven DCRGs and arachidonic acid pathway in COAD. **(A–H)** Western blots of the seven DCRGs in the SW480 cells treated with NG or HG. **(I–P)** Western blots of the seven DCRGs in the NCM460 cells treated with NG or HG. **(Q–S)** ELISA of *PCGE2*, *LTC4*, and *LTB4* in the SW480 and NCM460 cells treated with NG or HG. * *P* < 0.05 compared to controls. HG, high glucose; NG, normal glucose.

To verify the expression of the DCRGs and arachidonic acid metabolic pathways in tumors, we stimulated colon cancer and normal intestinal epithelial cells with high glucose and physiological glucose levels, respectively. All seven DCRGs were significantly elevated in the colon cancer epithelial cells under a high glucose environment (**Figures 7A–H**). However, in normal intestinal epithelial cells, there was no significant difference in the expression of the DCRGs under a normal or high glucose environment (**Figures 7I–P**). These results suggest that high glucose stimulation can lead to increased expression of the seven DCRGs in colon cancer epithelium, while it does not affect their expression in normal colon epithelial cells.

For the arachidonic acid pathway, the expression of *PEG2* in colon cancer epithelial cells was significantly increased under high glucose, while *LTC4* levels were unaltered compared to the controls. *PEG2* was not significantly different in normal intestinal epithelial cells, while *LTC4* expression was significantly higher under high glucose stimulation. Moreover, *LTB4* did not exhibit significant differences in either colon cancer or normal intestinal epithelial cells (**Figures 7Q–S**).

## Discussion

**Figure 8:**
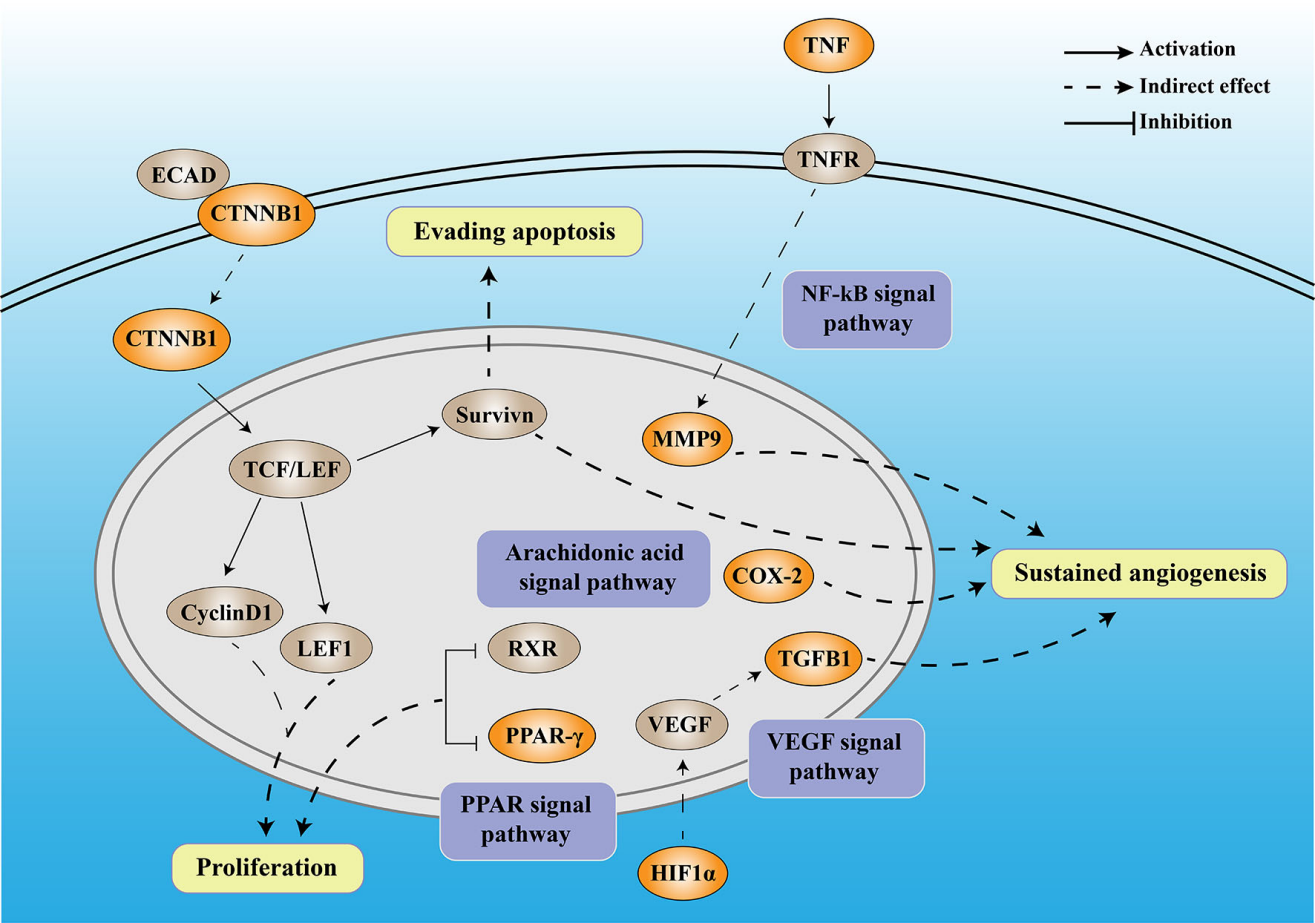
The mechanistic diagram of diabetes-based cancer-associated inflammation network (DCIN). The legend is included in the upper right corner of the image. The double-framed line in the upper part of the picture represents the cell membrane, and the double-framed line in the middle circle represents the nucleus. The orange gene represents the DCRGS screened out in this study, while the gray gene represents an important biological factor.

Numerous studies have shown that DM can promote the occurrence and development of tumors through inflammation. In this study, through bioinformatics analysis, we screened seven DCRGs, including *PPARG*, *MMP9*, *CTNNB1*, *TNF*, *TGFB1*, *PTGS2*, and *HIF1A*, and established a DCIN to comprehensively understand the diabetes-inflammation-cancer interaction (**Figure 8**). In addition, we found that the level of immune infiltration, immune subtypes, tumor microenvironment, mutation, the correlation with TMB and MSI, and the sensitivity of immune checkpoint-related drugs differed in this DCIN. Furthermore, the expression of DCRGs and the arachidonic acid metabolic pathway was verified *in vitro*. Together, we defined a DCIN and illustrated the potential mechanism through which diabetes may influence cancer.

The DCIN is composed of seven DCRGs with established internal connections, supporting the notion of its existence. Among DCIN, tumor necrosis factor (TNF) is a pro-inflammatory cytokine produced and secreted by cytotoxic lymphocytes upon tumor target recognition. Matrix metalloproteinases 9 *(MMP9)*, a member of the MMP family, has been widely studied in various cancers due to its critical role in the breakdown and reconstruction of the extracellular matrix during colorectal cancer (CRC) invasion and metastasis (29, 30). Both the tumor cells and surrounding stromal cells can synthesize MMPs (31). TNF-α upregulates *MMP9* expression *via* c-Src, MAPKs, and NF-κB pathways (32). Moreover, *MMP9* regulates the vascular endothelial growth factor (VEGF) signaling axis by cleaving membrane-bound VEGF, resulting in increased bioavailability of its receptor, VEGFR2 (33). Our results showed that *TNF* was highly expressed in almost all tumors, confirming its carcinogenicity, whereas *MMP9* was highly expressed in all tumors except THYM, suggesting its potential utility as a therapeutic target for various tumors.

β-Catenin (*CTNNB1*) is a multifunctional protein involved in transcription and cell adhesion (34) that affects tumors in two ways. On the one hand, *CTNNB1* promotes tumor development and progression through Survivin, which inhibits apoptosis, promotes cell cycle progression, and enhances angiogenesis (35). On the other hand, *CTNNB1* knockdown inhibits the Wnt/β-catenin signaling pathway and downregulates the expression of downstream genes, including axin 2, lymphoid enhancer-binding factor 1 (*LEF1*), and cyclin D1, thereby inhibiting tumor proliferation (36). In our study, *CTNNB1* was highly expressed in all the tumors except CESC, OV, UCEC, and UCS, confirming its carcinogenicity.

*HIF-1α* is part of the Hypoxia-inducible factor-1 (HIF-1) family (37), which is a key factor regulating cell adaptation to hypoxia (38). HIF1 can induce the expression of several pro-angiogenic factors, including vascular endothelial growth factor (VEGF) and VEGF receptors, FLT-1 and FLK-1. Among all these pro-angiogenic factors, VEGF-A, a potent endothelial mitogen, is a notable protein since it is highly expressed in many human tumors (39, 40). However, during the later stages of tumor development, *TGFβ1* functions as a tumor promoter by inducing the epithelial-mesenchymal transition (EMT) in cancer cells, resulting in increased invasion and metastasis (41). Meanwhile, *PTGS2*, also known as *COX-2*, contributes to angiogenesis (42). *COX-2* overexpression in colon cancer cells leads to the production of prostaglandins and the induction of pro-angiogenic factors such as vascular endothelial growth factor (VEGF) and basic fibroblast growth factor (bFGF), stimulating endothelial cell migration and tube formation (43, 44). In our study, the high and low expression levels of *TGFβ1* in different tumors exhibited significant differences, which also reflected the dual role of *TGFβ1*. Peroxisome proliferator-activated receptors (PPARs) comprise three isotypes: *PPARα, PPARγ, and PPARδ*. The *PPARG* gene encodes *PPARγ* (45). The anti-tumor functions of *PPARα* and *PPARγ* are currently inconclusive and controversial (46). Downregulation of *PPARγ* could inhibit the proliferation of T24 cells, which might be caused by cell cycle arrest in the G0/G1 phase (47). *PPARG* has been demonstrated to have anti-neoplastic effects by arresting the cell cycle, causing terminal differentiation, and inhibiting inflammation (48). Here, over-expression of *PPARG* was a protective factor for five tumors and a risk factor for another five. It also confirmed that the role of *PPARG* varies in the different cancer types. Moreover, we found that high glucose stimulation can lead to increased expression of the seven DCRGs in colon cancer epithelium *in vitro*, while it does not affect their expression in normal colon epithelial cells. These results suggest that DM may promote the occurrence and development of tumors through DCIN. Together with the previous studies, our established DCIN revealed the internal relationship of each DCRG. We also verified the expression of DCRGs *in vitro*.

Meanwhile, to further verify the interaction between DCRGs, we analyzed them from different perspectives. The GSVA analysis showed that DCRGs were closely related to the arachidonic acid metabolic pathway. Arachidonic acid, specifically its metabolites, has attracted much attention in cancer biology, especially in inflammation (49). The importance of arachidonic acid in biology lies in the fact that it can be metabolized by three different enzyme systems, cyclooxygenases (COXs, also referred to as PGG/H synthases), lipoxygenases (LOXs), and cytochrome P450 (CYP) enzymes (ω-hydroxylases and epoxygenases) to generate an impressive spectrum of biologically active fatty acid mediators (50). The signaling of cyclooxygenase 2-prostaglandin E2-prostaglandin E2 receptors (COX-2-PGE2-EPs) is the central inflammatory pathway involved in carcinogenesis (51). COX is the primary enzyme in the synthesis of eicosanoids and exists in two isoforms: COX-1, which is ubiquitously expressed (52), and COX-2, which is expressed predominantly in inflammatory cells and upregulated in chronic and acute inflammations (53). Prostaglandins derived from COX-2 contribute to cancer progression and metastasis (54). The COX-2 expression is stimulated by different growth factors, cytokines, and prostaglandins, which are associated with inflammatory responses and have been shown as prognostic factors for malignancy (55). Furthermore, upregulation of COX-2 and PGE2 has been identified in many human cancers and precancerous lesions, and COX-inhibitory drugs have shown protective effects in colorectal cancer (56, 57). In addition to colorectal cancer, nonsteroidal anti-inflammatory drugs (NSAIDs) have also been associated with a reduced risk of breast, esophageal, stomach, bladder, ovary, and lung cancers (58, 59). We showed that the expression of PEG2 in colon cancer epithelial cells was significantly increased under high glucose compared to the control. In contrast, PEG2 had no significant difference in normal intestinal epithelial cells. These results suggest that DM may promote cancer occurrence and development through the arachidonic acid metabolic pathway.

In the immune infiltration analysis of the seven DCRGs and 26 immune cells, we found that *MMP9* was associated with 12 tumors, *PTGS2* was associated with 7 tumors, and *TNF* was associated with 5 tumors. There was a positive correlation between *MMP9* and all the immune cells. These results suggest that DCRGs may influence tumor development and prognosis through the immune cells. Our genetic analysis showed a high frequency of copy number variations of DCRGs, and the prognosis of the mutational group was lower than the non-alterative group, indicating that mutations could affect the occurrence and development of tumors. According to research, breast cancer has been linked to mutations in *MMP9* (60). Additionally, it may promote the invasion and metastasis of bladder cancer (61). *PPARG* mutations are closely associated with digestive tract cancers (colon, stomach, esophagus, and pancreas), melanoma, breast cancer, prostate cancer, and bladder cancer (62). The role of these seven DCRGs mutations in different tumors needs further investigation. Moreover, our analysis suggested that the TMB and MSI are correlated with the expression of the seven DCRGs in most tumors. In TNYM, a positive correlation was observed between the expression of all DCRGs and both TMB and MSI. However, in all DCRGs except THYM, a negative correlation was observed between TMB/MSI and TNF expression, indicating that the treatment strategies for TNYM may differ from other cancers at the level of immune checkpoints. In addition, drug sensitivity analysis suggested that the seven DCRGs potentially affected tumor drug response. Among them, Nelarabine was highly correlated with *TNF*, which has guiding clinical significance for the selection of anti-tumor therapy. Our analysis of immune subtypes revealed that DCRGs, in addition to CTNNB1, exhibited a strong association with the C6 immune subtype. The C6 is the immune subtype characterized by TGF-β dominance, which suggests that DCRs might be closely associated with the B-transforming growth factor. This finding is consistent with our previous results.

To further investigate the role of the seven DCRGs in colon cancer cells, single-cell sequencing data of colon cancer were analyzed. As a result, the activity of the seven DCRGs, estimated by the AUC value of each cell, was highest in macrophages. A previous study has reported that macrophages can promote pro-tumor inflammation by secreting pro-inflammatory cytokines. Macrophages can induce immune responses and support tumor growth and survival of malignant cells (63). Macrophages also play diverse roles in cancer development, ranging from anti-tumor activity at early stages of progression to tumor promotion in the most commonly established cancers (64). For DM, it has previously been suggested that hyperglycemia could enhance cancer immune evasion by increasing O-GlcNAcylation to induce alternative macrophage polarization (65). Thus, macrophages play a crucial role in the development of DM and cancer. *MMP9* is secreted by macrophages and acts on PAR1 of PDAC cells to induce epithelial-to-mesenchymal transition. This macrophage-induced mesenchymal transition supports the tumor-promoting effect of macrophage influx, explaining the dual contribution of these immune cells to tumor growth (66). Studies have shown that TNF-α regulates diabetic macrophage function through the histone acetyltransferase MOF (67). Therefore, based on the single-cell and our experimental validation results, we concluded that the seven DCRGs were associated with immune inflammation, especially macrophages.

This study proposes the novel concept of DCIN, but it still has some limitations that need to be addressed. Firstly, the study only includes samples from China, which may limit the generalizability of the prediction model to other populations. Secondly, the results of this study have not been verified by other independent databases. However, we have followed up the results with our molecular biology experiments to make the results more convincing. Finally, the analysis in this study focused on the correlation between the DCRGs; however, biostatistical correlations alone cannot elucidate direct interactions and regulatory mechanisms. Therefore, our future experiments aim to verify the interaction of various molecules in the DCIN and elucidate the potential mechanisms of molecular action on tumors.

## Conclusion

In this study, we constructed a DCIN for the first time (see **Figure 8**), which has provided valuable insights into the potential mechanisms by which T2DM may contribute to tumor occurrence and development. While additional research is needed to confirm our findings, our study results have also offered promising leads for the development of new therapeutic strategies aimed at preventing and treating malignant tumors.

## Data availability statement

The raw data supporting the conclusions of this article will be made available by the authors, without undue reservation.

## Author contributions

WP designed this study and revised the manuscript. YT and JK collected the data and performed the bioinformatic and statistical analysis, figure visualization, and manuscript writing. HL conducted WB and ELISA experiments. YL, AZ, RH, ZZ, and XC revised the manuscript. All authors contributed to the article and approved the submitted version. 

## Supplementary material

Supplementary Figure 1 The gene set variation analysis (GSVA) pathways of the seven DCRGs. The length of the bar represents the t value, the blue bar represents t > 2, and the green bar represents t< -2

Supplementary Figure 2 The survival analysis of the seven DCRGs across cancers. Kaplan-Meier plots of DCRGs across cancers show the differential survival outcomes of high- and low-expressing DCRGs (*P* < 0.05).

Supplementary Figure 3 The correlation between immune cell infiltration and the seven DCRGs across cancers. The correlation coefficient R and the *P*-values are presented in the upper left corner of each panel. Only results for *P*<0.5 are shown.

Supplementary Figure 4 Correlation analysis of the DCRGs with the stemness indices and microenvironment scores. **(A, B)** Heatmap showing the correlation of the seven DCGRs’ expression with immune and stromal scores. **(C, D)** Heatmaps showing the correlation of the DCRGs’ expression with stemness indices (DNAss and RNAss) in diabetes-inflammation-cancer-network TCGA cancer types. DNAss: DNA methylation-based stemness score, RNAss: RNA-based stemness score. Red points represent a positive correlation, while blue points represent a negative correlation.

Supplementary Figure 5 Correlations of the seven DCRGs with the immunomodulators, TMB and MSI. **(A)** Correlations between expression of the seven DCRGs and tumor mutation burden (TMB) across cancers. **(B)** Correlations between expression of the seven DCRGs and microsatellite instability (MSI) across cancers. **(C)** Correlations between expression of the seven DCRGs and the immunomodulators in COAD.

Supplementary Figure 6 Drug response analysis. The correlation between drug sensitivity and the DCRGs across different cancers from the TCGA database. The scatter plots are ranked by *p*-values.

Supplementary Figure 7
**(A)** Gene set variation analysis of metabolic pathway in macrophages between normal and adenoma tissues. **(B)** Gene set variation analysis of metabolic pathway in macrophages between normal and carcinoma tissues. **(C)** UMAP plot of the AUC score of each cell type.

## Glossary

**Table:**

ACC	Adrenocortical carcinoma
BLCA	bladder urothelial carcinoma
BRCA	breast invasive carcinoma
CESC	cervical squamous cell, carcinoma and endocervical adenocarcinoma
CHOL	cholangiocarcinoma
COAD	colon adenocarcinoma
DCIN	diabetes-based cancer-associated inflammation network
DCRGs	diabetes and cancer-related genes
DLBC	lymphoid neoplasm diffuse large B-cell lymphoma
DM	diabetes mellitus
ESCA	esophageal carcinoma
GBM	glioblastoma multiforme
HNSC	Head and neck squamous cell carcinoma
KICH	kidney chromophobe
KIRC	kidney renal clear cell carcinoma
KIRP	kidney renal papillary cell carcinoma;
LAML	acute myeloid leukemia
LGG	brain lower grade glioma
LIHC	Liver hepatocellular carcinoma
LUAD	lung adenocarcinoma
LUSC	lung squamous cell carcinoma
MESO	mesothelioma
OV	ovarian serous cystadenocarcinoma;
PAAD	pancreatic adenocarcinoma
PCPG	pheochromocytoma and paraganglioma
PRAD	prostate adenocarcinoma
READ	Rectum adenocarcinoma
SARC	sarcoma
SKCM	skin cutaneous melanoma
STAD	stomach adenocarcinoma
TGCT	testicular germ cell tumors
THCA	Thyroid carcinoma
THYM	thymoma
UCEC	uterine corpus endometrial carcinoma;
UCS	uterine carcinosarcoma
UVM	uveal melanoma.
