# Supplementary figures and images for: Diabetes mellitus induces a novel inflammatory network involving cancer progression: Insights from bioinformatic analysis and in vitro validation
Source: Front Immunol. 2023 Mar 23;14:1149810. doi: 10.3389/fimmu.2023.1149810 (PMC10076825; doi:10.3389/fimmu.2023.1149810)

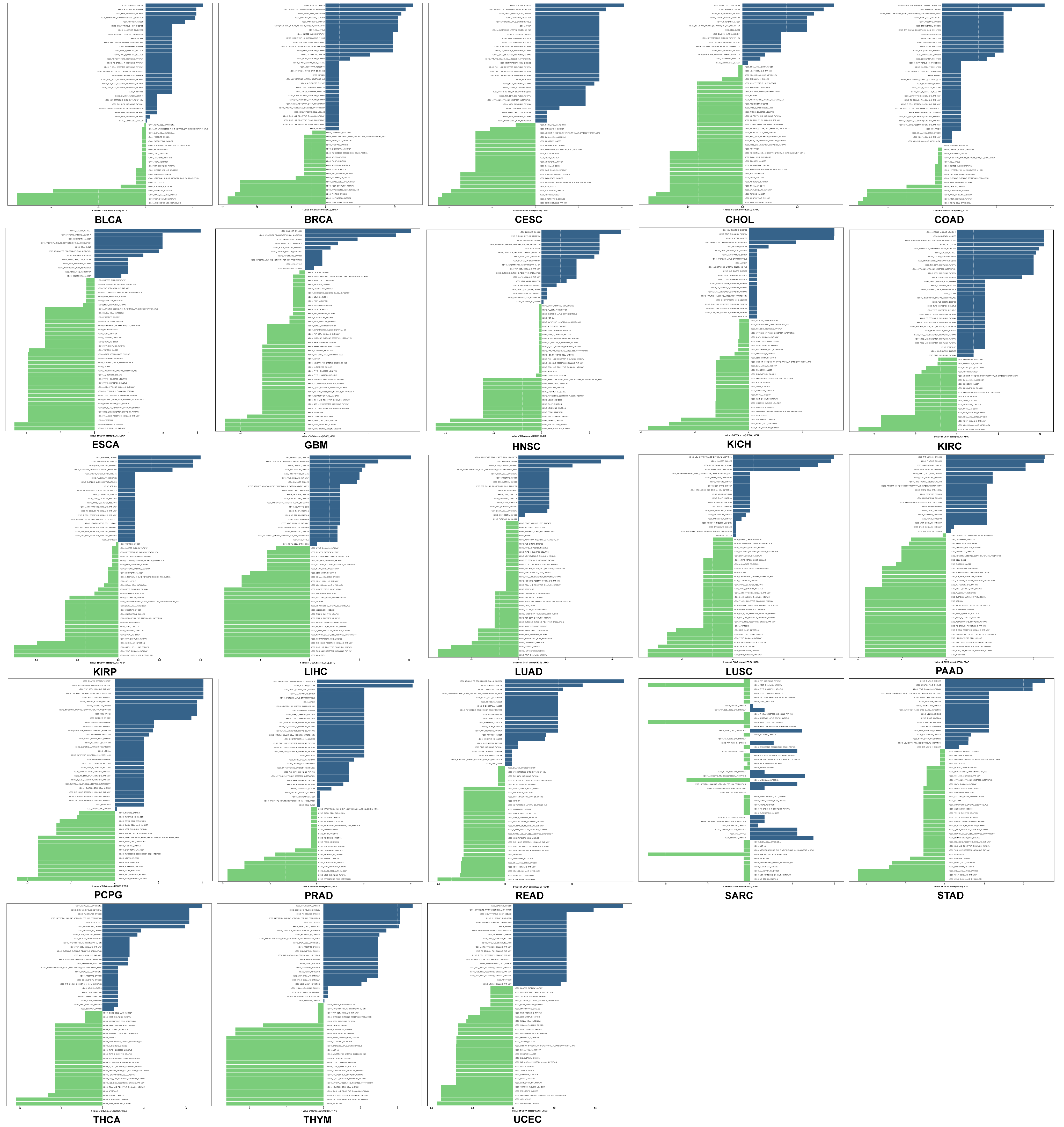

Supplement: Supplementary Figure 1 — The gene set variation analysis (GSVA) pathways of the seven DCRGs. The length of the bar represents the t value, the blue bar represents t > 2, and the green bar represents t< -2 [file Image_1.tif]

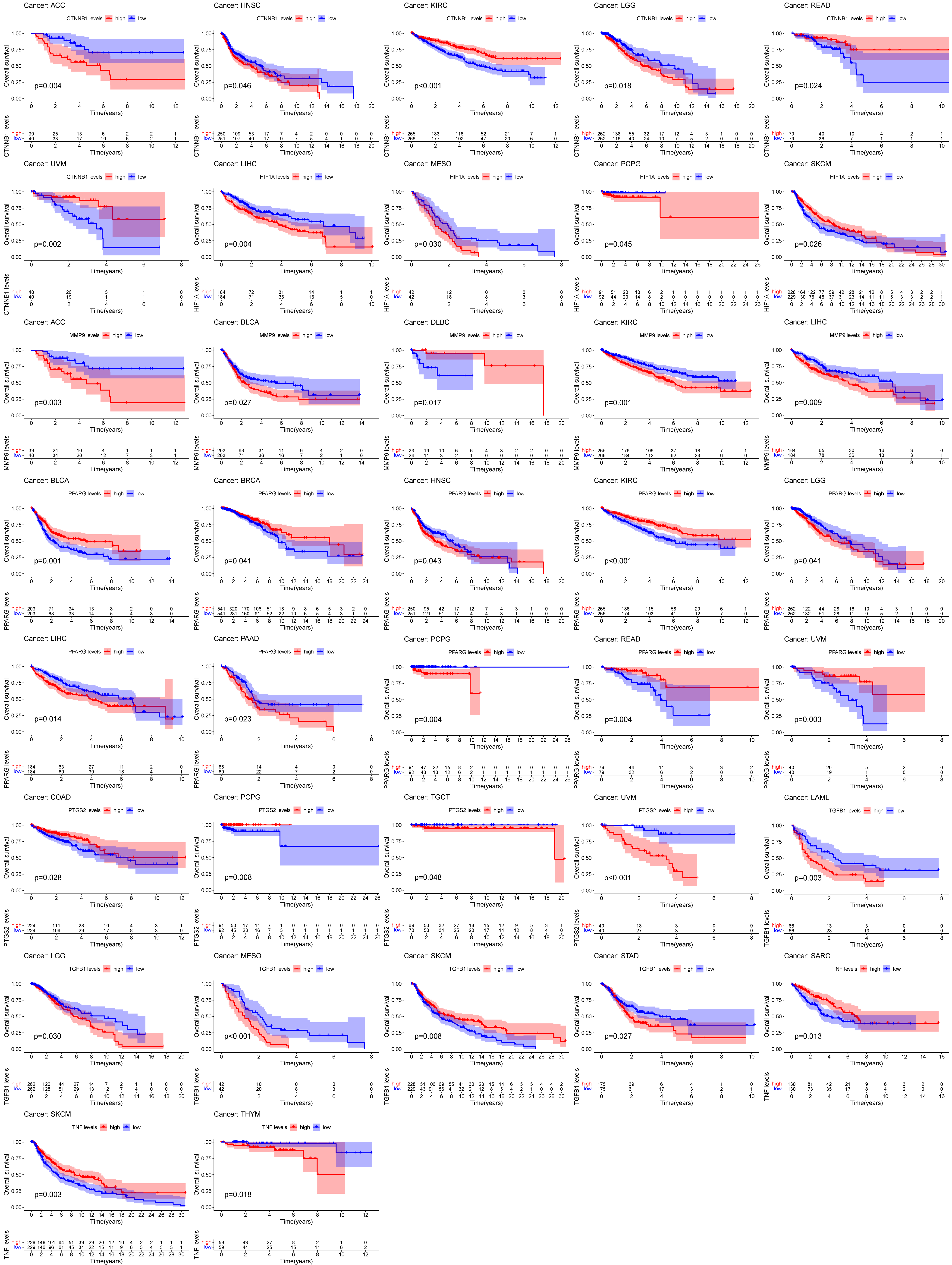

Supplement: Supplementary Figure 2 — The survival analysis of the seven DCRGs across cancers. Kaplan-Meier plots of DCRGs across cancers show the differential survival outcomes of high- and low-expressing DCRGs (P < 0.05). [file Image_2.tif]

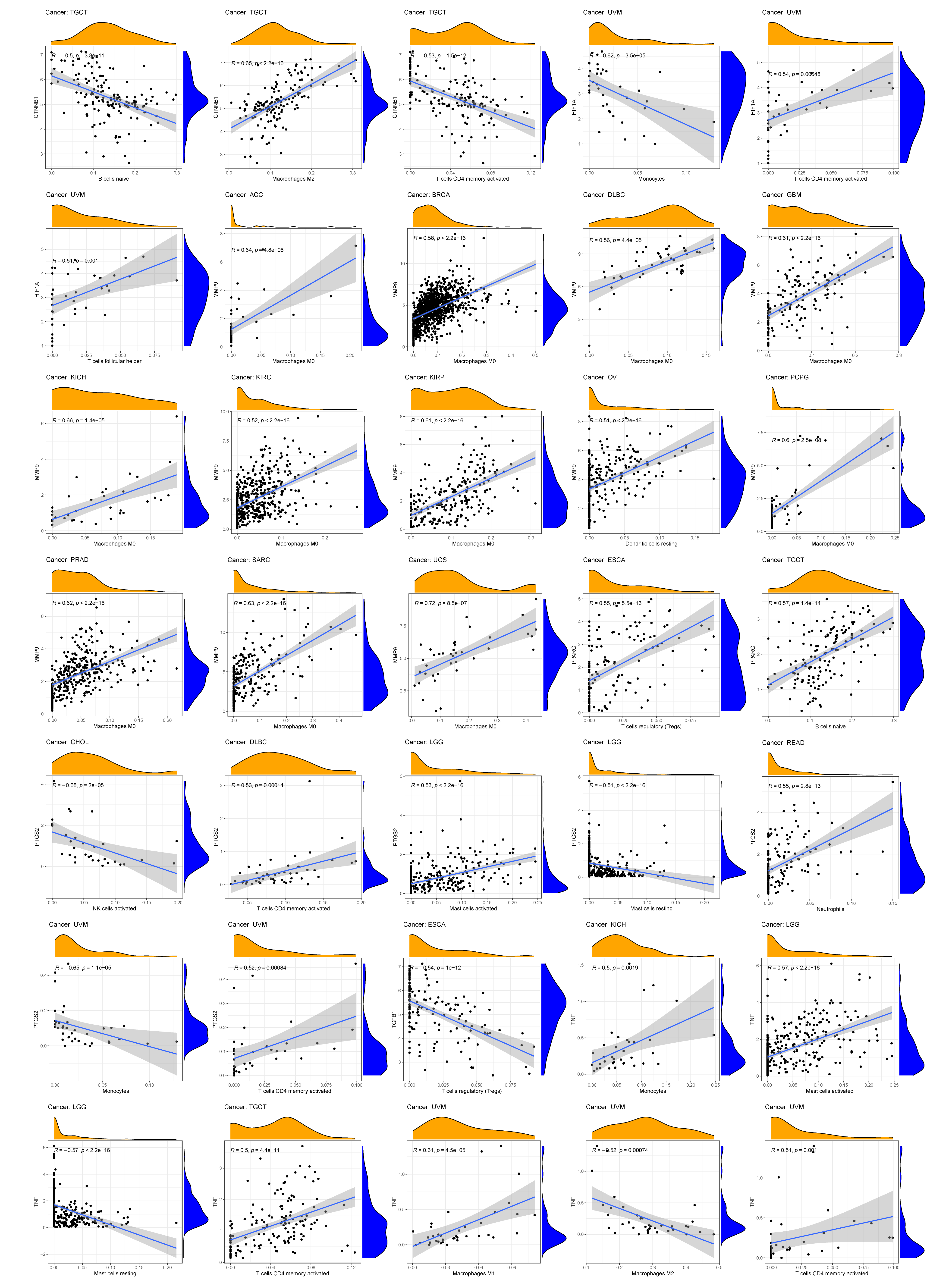

Supplement: Supplementary Figure 3 — The correlation between immune cell infiltration and the seven DCRGs across cancers. The correlation coefficient R and the P-values are presented in the upper left corner of each panel. Only results for P<0.5 are shown. [file Image_3.tif]

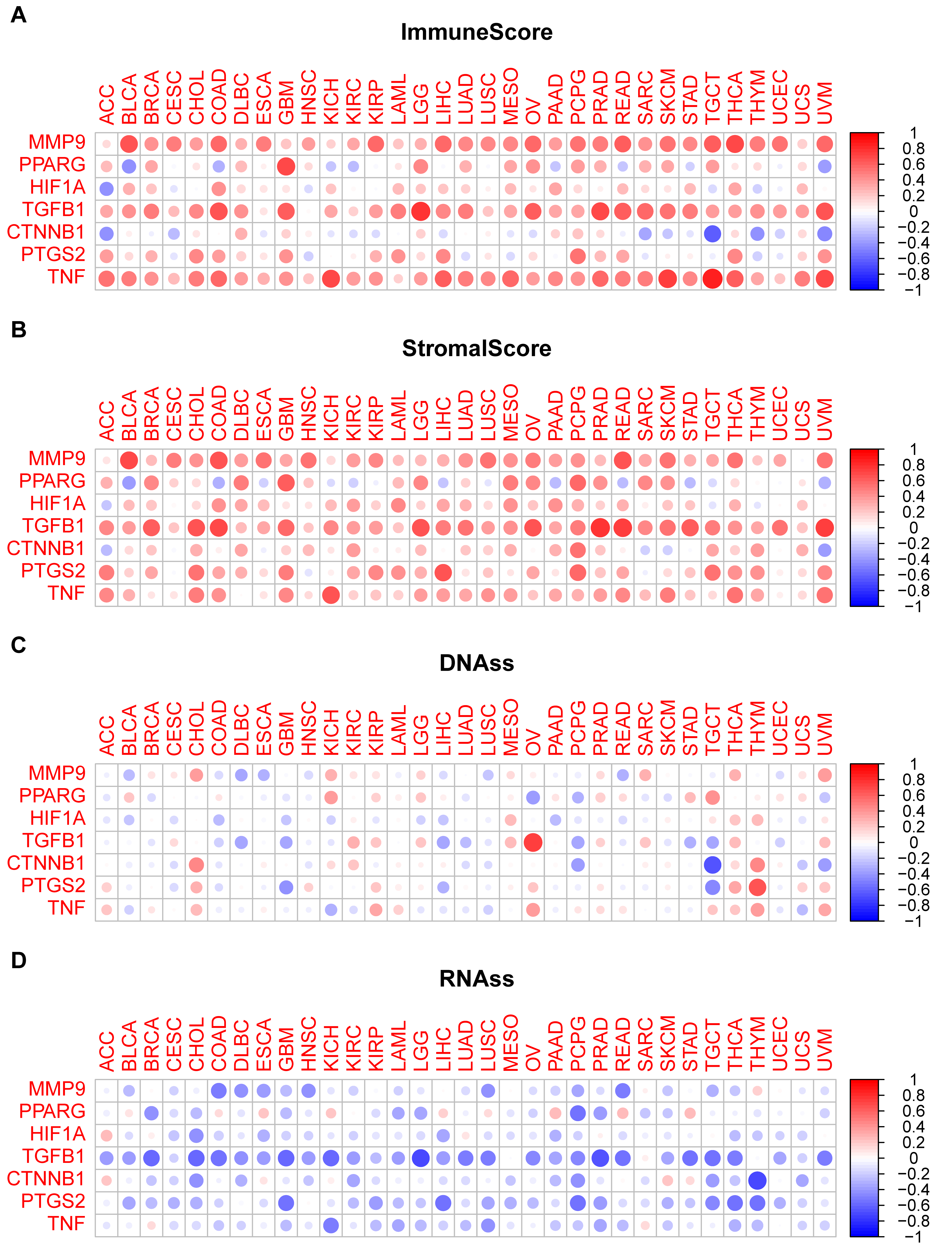

Supplement: Supplementary Figure 4 — Correlation analysis of the DCRGs with the stemness indices and microenvironment scores. (A, B) Heatmap showing the correlation of the seven DCGRs’ expression with immune and stromal scores. (C, D) Heatmaps showing the correlation of the DCRGs’ expression with stemness indices (DNAss and RNAss) in diabetes-inflammation-cancer-network TCGA cancer types. DNAss: DNA methylation-based stemness score, RNAss: RNA-based stemness score. Red points represent a positive correlation, while blue points represent a negative correlation. [file Image_4.tif]

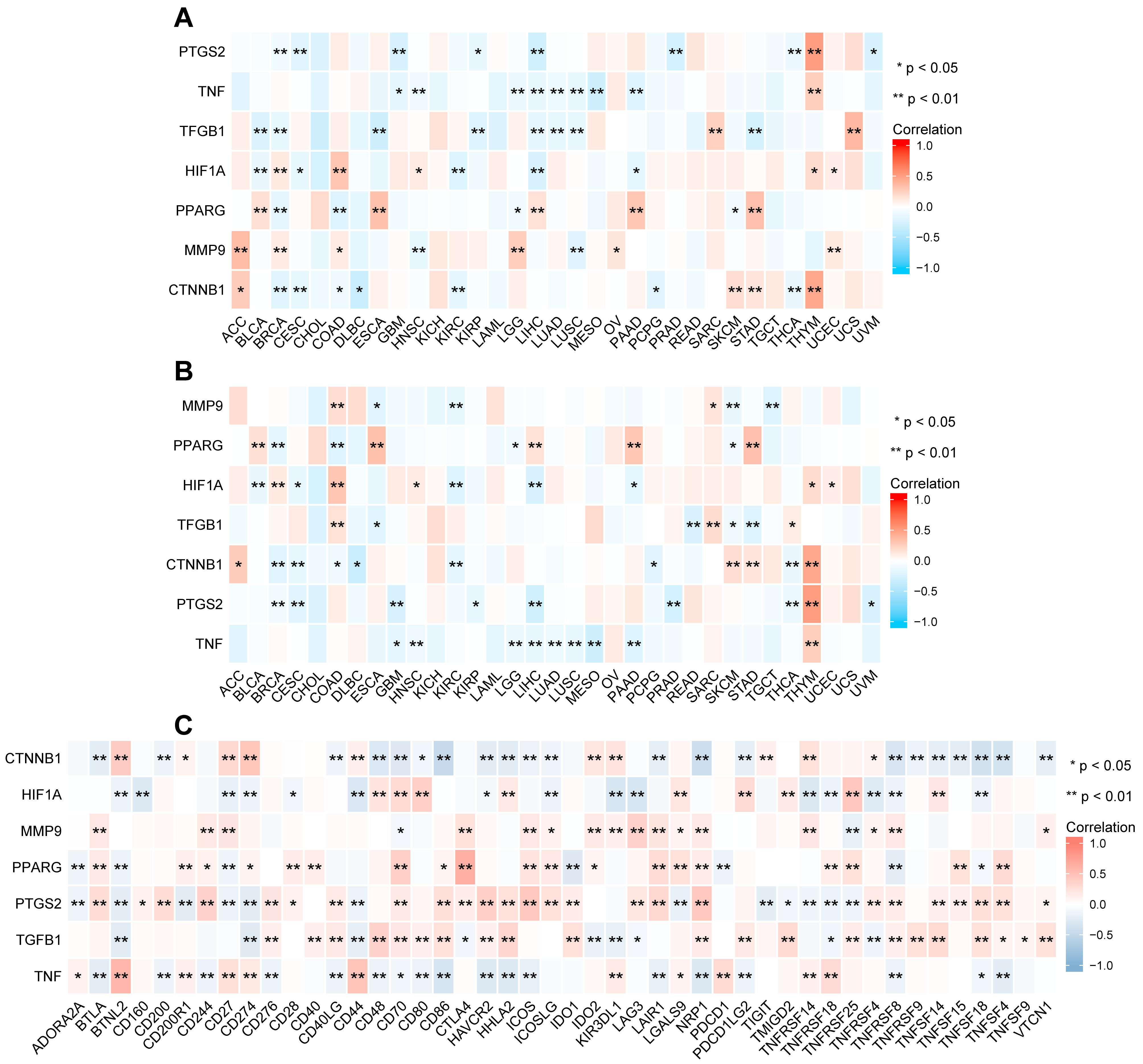

Supplement: Supplementary Figure 5 — Correlations of the seven DCRGs with the immunomodulators, TMB and MSI. (A) Correlations between expression of the seven DCRGs and tumor mutation burden (TMB) across cancers. (B) Correlations between expression of the seven DCRGs and microsatellite instability (MSI) across cancers. (C) Correlations between expression of the seven DCRGs and the immunomodulators in COAD. [file Image_5.tif]

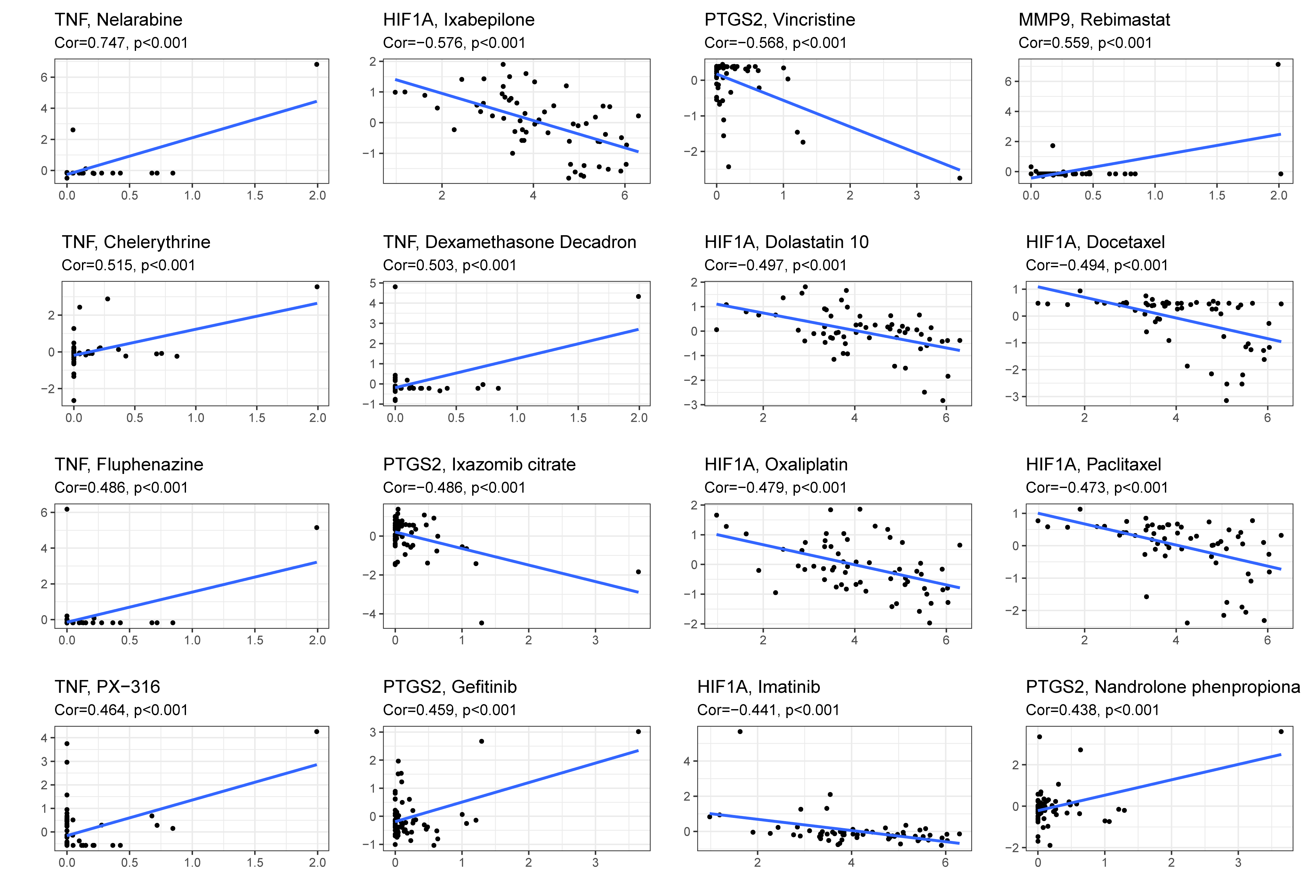

Supplement: Supplementary Figure 6 — Drug response analysis. The correlation between drug sensitivity and the DCRGs across different cancers from the TCGA database. The scatter plots are ranked by p-values. [file Image_6.tif]

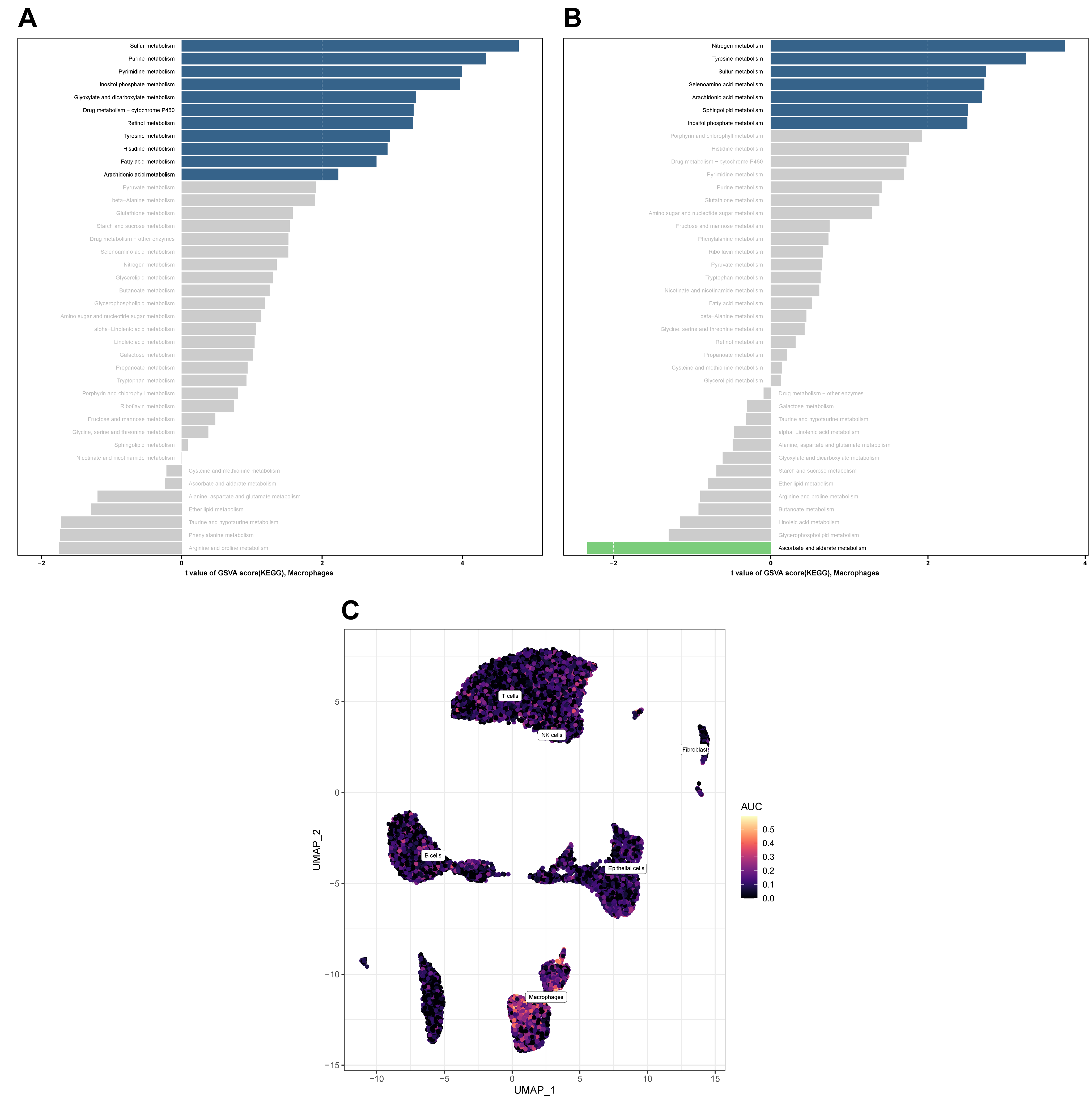

Supplement: Supplementary Figure 7 — (A) Gene set variation analysis of metabolic pathway in macrophages between normal and adenoma tissues. (B) Gene set variation analysis of metabolic pathway in macrophages between normal and carcinoma tissues. (C) UMAP plot of the AUC score of each cell type. [file Image_7.tif]
